# Improved YOLOv5 infrared tank target detection method under ground background

**DOI:** 10.1038/s41598-023-33552-x

**Published:** 2023-04-17

**Authors:** Chao Liang, Zhengang Yan, Meng Ren, Jiangpeng Wu, Liping Tian, Xuan Guo, Jie Li

**Affiliations:** 1 School of Artificial Intelligence, Xidian University, Xi’an, 710071 China; 2 Xi’an Modern Control Technology Research Institute, Xi’an, 710065 China

**Keywords:** Engineering, Electrical and electronic engineering

## Abstract

The detection precision of infrared seeker directly affects the guidance precision of infrared guidance system. To solve the problem of low target detection accuracy caused by the change of imaging scale, complex ground background and inconspicuous infrared target characteristics when infrared image seeker detects ground tank targets. In this paper, a You Only Look Once, Transform Head Squeeze-and-Excitation (YOLOv5s-THSE) model is proposed based on the YOLOv5s model. A multi-head attention mechanism is added to the backbone and neck of the network, and deeper target features are extracted using the multi-head attention mechanism. The Cross Stage Partial, Squeeze-and-Exclusion module is added to the neck of the network to suppress the complex background and make the model pay more attention to the target. A small object detection head is introduced into the head of the network, and the CIoU loss function is used in the model to improve the detection accuracy of small objects and obtain more stable training regression. Through these several improvement measures, the background of the infrared target is suppressed, and the detection ability of infrared tank targets is improved. Experiments on infrared tank target datasets show that our proposed model can effectively improve the detection performance of infrared tank targets under ground background compared with existing methods, such as YOLOv5s, YOLOv5s + SE, and YOLOV 5 s + Convective Block Attention Module.

## Introduction

In complex ground background, the grayscale characteristics of infrared tank targets are very similar to the background, which is easily influenced by background factors, making the target characteristics inconspicuous^1^. Moreover, when infrared seekers detect targets at a long distance, the targets in infrared images are often weak targets, the target scale changes greatly, and environmental interference factors are many^2,3^. Therefore, the seeker needs to find and detect infrared tank targets quickly from the complex battlefield environment to deal with complex, changeable situations more quickly.

Compared with visible light images, infrared images are depicted by thermal radiation, which has the characteristics of long-acting distance and can be used day and night^4,5^ . However, infrared images also have the characteristics of high noise and poor spatial resolution, which presents higher requirements for long-distance target detection, especially in ground background^6^. Traditional methods use the gray feature method^7,8^, the spatial filtering method^9,10^, the Markov random field method^11–13^, and the wavelet transform method^14–17^. However, these methods have some problems, such as requiring prior conditions, artificially defining features, high false alarm rate^18–22^, and inability to suppress the background to infrared targets. With the development of artificial intelligence technology, the convolutional neural network (CNN) can extract deeper image features, realize end-to-end learning, and obtain better image interpretation ability^23–25^. The methods based on CNN are mainly divided into two stages and one stage. The two-stage methods include Region-CNN (R-CNN)^26^, FAST R-CNN^27^ [27], and FAST R-CNN^28^. The one-stage methods include single-shot multi-box detector^29^ and YOLO^30^. Based on the YOLO method, YOLO9000^31^, YOLOv3^32^, YOLOv4^33^, YOLOv5^34^, and other methods have been developed. Although the two-stage method has high accuracy, it has a large amount of calculation and slow detection speed, whereas the one-stage method has high speed but low accuracy^35,36^. When an infrared seeker detects at a long distance, the target is far, the target pixels are few, and suppressing the ground background and focusing on the identified target is necessary. Moreover, due to the limitation of hardware resources, adopting a lighter network is necessary. The different network configurations of YOLOv5 model are divided into YOLOV5^37^, YOLOv5m^38^, YOLOv5l^39^, and other structures, among which YOLOV5 model has the simplest structure, so it is considered.

With the appearance of the above detection methods, some researchers have done a lot of research on infrared target detection under complex background. In this part, we briefly review the related work of previous researchers. The related work of infrared target detection is mainly elaborated through two aspects, the traditional method and the method of convolutional neural network.

In terms of traditional methods, Gao et al. modeled the infrared target detection problem based on Mixture of Gaussians (MoG) and Markov Random field (MRF) method, and proposed a MRF guided MoG noise model under Bayesian framework to realize target detection under complex background^40^. Xie et al.^41^proposed a method of local average gray difference measurement (LAGDM) to highlight the difference between infrared target and background, and then generated a LAGDM map to enhance target features and suppress clutter. This method has good robustness to cloudy sky, sea and sky, mountains and forests and other backgrounds. Zhang et al.^42^ proposed an infrared target detection method based on morphology and wavelet transform, which suppresses clutter and performs wavelet decomposition, extracts high-frequency components and performs adaptive fusion to improve detection rate and reduce false alarm rate. Luo et al.^43^proposed an adaptive spatial filtering algorithm, which can effectively detect small infrared targets in undulating background with little loss of target information. Aiming at the problem of infrared target recognition in desert background, Li et al.^44^ proposed an improved kernel fuzzy clustering algorithm to cluster infrared images and polarized images to distinguish the target from the background.

In terms of convolutional neural networks, Ding et al. improved the detection accuracy of infrared targets by discarding the low-resolution layer and enhancing the high-resolution layer based on the SSD model^45^. Based on the convolutional neural network CNN, Du et al. designed effective small anchors in the shallow layer of ResNet50 to improve the detection accuracy of infrared small targets^46^. Ou et al.^47^ used an improved faster R-CNN target detection model to improve the feature extraction network based on VGG16 to improve the detection accuracy of infrared targets in complex backgrounds. Based on the YOLOv4 model, Du et al., proposed an FA-YOLO model, which adds a dilated convolutional block attention module to CSPdarknet53 in the backbone, and proposes a negative sample focusing mechanism to reduce the influence of useless complex background information on detection results, and improve the vehicle detection in remote sensing images with complex backgrounds^48^.

### Motivation

The infrared seeker needs to detect the target in real-time during the working process. However, in the actual flight process, there are some problems, such as less feature information, fast scale change and complex ground background of the infrared target. In order to improve the guidance accuracy of the seeker, we need the seeker to have the ability to detect the target accurately and suppress the unfavorable factors of the complex ground background on the target detection.

In this paper, the YOLOv5s-THSE model is proposed to solve the problem of infrared seeker target detection in the ground background. Firstly, aiming at the problem of less feature information of infrared targets, we add a multi-head attention mechanism to the backbone and neck of YOLOv5s model to improve the network model's ability to mine the features in the whole image and make the extracted features more abundant. The complex and variable ground background can easily cause the false detection of the targets, leading to poor detection accuracy. Therefore, we add the CSP_SE module to the neck of the network to suppress the influence of the background on the target detection. The seeker is far away from the target in the initial stage, and the target is smaller than 16*16. In order to improve the detection accuracy of the seeker in the initial stage, a small target detection Head is introduced into the Head of the network to improve the detection accuracy of the seeker for small targets in the initial search stage, so as to improve the application range of the seeker. Through the above means, the recognition of the target itself was focused from multiple dimensions to suppress the ground background interference. On the other hand, in order to solve the problem that the predicted position is prone to deviate when the seeker is far away from the target, and to make the target frame more stable in the regression process when the target is small, In this paper, we change the GIoU (Intersection over Union) of the loss function in the YOLOv5s model to CIoU (Complete Cntersection over Union), which makes the target box more stable in the process of training and regression.

### Main contributions

The main contributions of this work are as follows:

1. The multi-head attention mechanism is introduced into the YOLOv5s network model to improve the feature extraction ability of the network model for infrared targets in the image.
2. The CSP_SE module is added to the YOLOv5s model, which aims to suppress the complex ground background and make the model pay more attention to the infrared target itself.
3. The small target detection head is added to the Head of YOLOv5s model, which deals with the small target detection problem faced in the initial stage of the seeker. On the other hand, the loss function uses CIoU, which can make the training of the network, especially the training regression of small targets, more stable and easy to converge the model.

### Organization of the Paper

The remainder of the text is arranged as follows: the second part introduces the proposed You Only Look Once, Transform Head Squeeze-and-Excitation model (YOLOv5s-THSE) model and then describes the network structure, including the improved small target detector, the CSP_SE model, the multi-head attention mechanism, the loss function, and the regression of the target box. The third part of this paper introduces the experimental data, compares the YOLOv5s-THSE model with the YOLOv5s, YOLOv5s + SE^49^, and YOLOv5s + CBAM^50^ models, and analyzes the experimental results. The last part presents the conclusion of this paper.

## Methods and models

### Network structure

**Figure 1:**
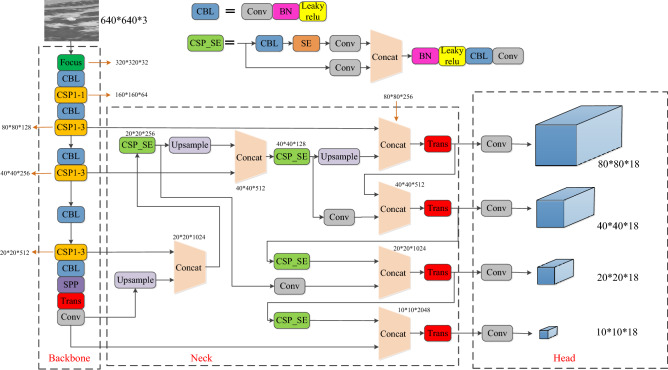
Improved YOLOv5s-THSE network structure diagram.

The YOLOv5 model performs well in detection speed, but its detection accuracy is low for infrared target recognition with complex ground background. Therefore, based on YOLOv5, this paper improves the network and proposes the YOLOv5-THSE model, which can effectively suppress the background and make it more accurate in detecting small targets. Its network structure is shown in Fig. 1.

According to the characteristics of seeker detecting complex ground targets, it is improved from three parts: backbone, neck, and head. To make the detection network pay more attention to the target itself and suppress the ground background, the CSP_SE module is used in the neck part. The CSP_SE module is an improvement of the original CSP module in YOLOv5s, with the purpose of using the Squeeze-and-Exclusion (SE) attention mechanism^51^ to increase the attention to the characteristics of the target itself. During the flight of the seeker, the target shows multiscale drastic changes in the infrared image of the seeker, and the seeker’s ability to detect small targets is low. To solve these two problems, an 80*80*18 small target detector is added to the network to cope with the multi-scale changes of targets and improve the detection ability of small targets. To mine the deeper image semantic information of the target in the complex ground background, inspired by Transformer, the multi-head attention trans module is introduced in the output part of the backbone and the head to mine the deeper image semantic information.

### Infrared dim and small target detection head

In the initial stage of seeker searching for the target, the target is a weak infrared target because of the long distance between seeker and target. Therefore, in the head part of the network model, a detector is added to detect small, weak infrared targets, which can cope with the drastic change of the target scale in the image during the flight of the seeker. Figure 1 shows the newly added 80*80*18 detector is a high-resolution feature map, which is more accurate in detecting small, weak targets. Through the four detection heads in the network structure, the target detection can be dealt with in different scales.

### CSP_SE model

The CSP structure in YOLOv5s can reduce the calculation of the model and improve the detection speed of the model^52^. However, infrared targets often have few features, and the CSP structure cannot effectively extract the features of infrared targets. To solve this problem, this paper proposes a CSP_SE model structure, which adds the SE attention mechanism to the CSP structure, so that the network can learn the important information of the target adaptively, thus enhancing the extraction effect of the target features.

**Figure 2:**
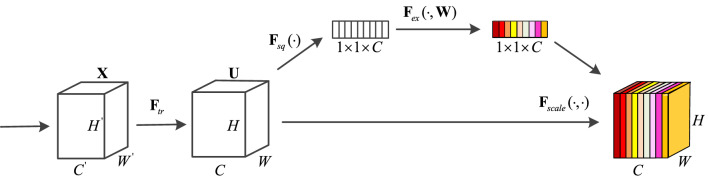
A squeeze-and-excitation block.

The structural diagram of the SE attention mechanism module is shown in Fig. 2. The SE model focuses on the relationship between channels, and the model can learn the weights of different channel features. The overall structure includes two steps: compression and excitation. Specifically, the SE model first maps X to the feature map U through the mapping ${\mathbf{F}}_{tr}$, then obtains a $1 \times 1 \times C$ feature map through pooling operation, and then calculates the $1 \times 1 \times C$ feature map through ${\mathbf{F}}_{ex} ( \cdot ,{\mathbf{W}})$ matrix to obtain the feature weight of $1 \times 1 \times C$. Finally, the weighted feature map can be obtained by fusion.

**Figure 3:**
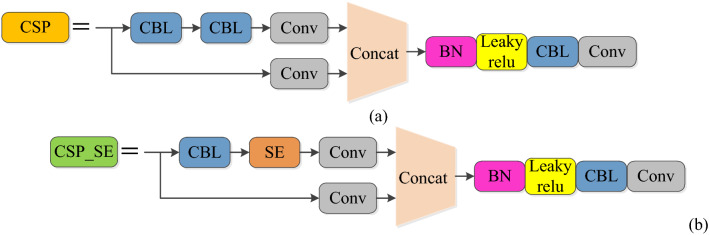
Comparison between CSP model and CSP_SE model. (**a**) CSP model, (**b**) CSP_SE model.

Figure 3 shows the CSP_SE model structure replaces a CBL module in the original CSP structure with an SE module, which can make the network pay more attention to the target.

### Multi-head attention mechanism module

**Figure 4:**
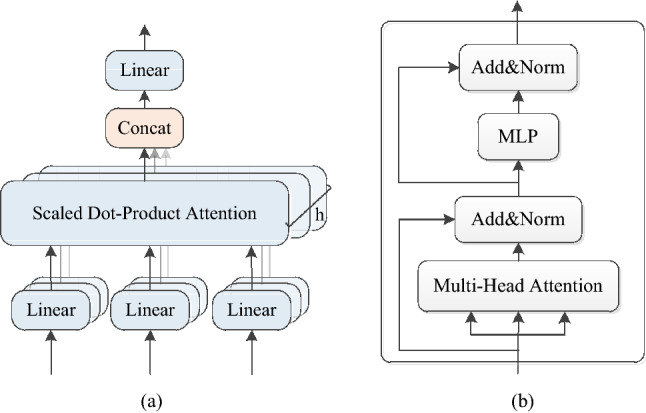
Structural diagram. (**a**) multi-head attention structure, (**b**) coding module in transformer.

In the process of seeker flying at high speed, the complex ground background easily affects target recognition. Inspired by the Transformer model^53^, this paper introduces the multi-head attention mechanism module into the network. When the single-headed self-attention mechanism model encodes the information of the current position, it will excessively focus on its own position and get an attention. Compared with single-headed attention, multi-headed attention transforms an input object into multiple features, and then calculates the self-attention of multiple features. Because each feature is independent in theory, it can also generate multiple independent attention, thus obtaining multi-dimensional image feature information. We use the coding module in the Transformer model to obtain deeper, richer semantic information to improve the adaptability of the network to the complex ground background. On the one hand, YOLOv5s-THSE adds the multi-head attention mechanism to the front end of the detector, replacing the CSP module in the original YOLOv5s network model, to make each detector capture richer global information and semantic information. On the other hand, in the backbone of the network, a CSP module is also replaced by a multi-head attention module. The purpose is to map the original features into multiple subspaces by using the multi-head attention mechanism, so that the network can extract image features from multiple dimensions. The multi-head attention mechanism module is mainly divided into two parts, as shown in Fig. 4. One is the multi-head attention mechanism layer, and the other is the multilayer perceptron. Each layer is connected in a cross-layer way.

### Loss function

The loss function in YOLOv5s model consists of three parts, namely, positioning loss, confidence loss, and category loss. Among them, confidence loss and category loss are calculated by cross-entropy, and the loss function is calculated as follows.

1 $$\begin{gathered} L{\text{oss}}_{conf} = - \sum\limits_{i = 0}^{K \times K} {I_{ij}^{obj} [\mathop {C_{i}^{j} }\limits^{ \wedge } \log C_{i}^{j} + (1 - \mathop {C_{i}^{j} }\limits^{ \wedge } )\log (1 - C_{i}^{j} )]} - \hfill \\ \lambda_{noobj} \sum\limits_{i = 0}^{K \times K} {\sum\limits_{j = 0}^{M} {I_{ij}^{noobj} [\mathop {C_{i}^{j} }\limits^{ \wedge } \log C_{i}^{j} + (1 - \mathop {C_{i}^{j} }\limits^{ \wedge } )\log (1 - C_{i}^{j} )]} } \hfill \\ \end{gathered}$$

2 $$Loss_{class} = - \sum\limits_{i = 0}^{K \times K} {I_{ij}^{obj} \sum\limits_{c \in classes} {\left[ {\mathop {P_{i}^{j} }\limits^{ \wedge } \log P_{i}^{j} + (1 - \mathop {P_{i}^{j} }\limits^{ \wedge } )\log (1 - P_{i}^{j} )} \right]} }$$

3 $$Loss_{object} = Loss_{loc} + Loss_{conf} + Loss_{class}$$

4 $$Loss_{loc} = 1 - GIoU$$

where *K* indicates that the feature map finally output by the network is divided into *K* × *K* grids, m indicates the number of anchor boxes corresponding to each grid, $I_{ij}^{obj}$ indicates the anchor box with target, $I_{ij}^{noobj}$ indicates the anchor box without target, and $\lambda_{noobj}$ indicates the confidence loss weight coefficient without target anchor box. The object detection problem faced by the model in this paper is a typical problem of an unbalanced number of categories. . Among K*K grids, there are usually only about three or four grids containing objects, and even fewer small targets, and the rest are all grids without objects. If the weights in the loss function are not set, the mAP of object detection will not be too high, because the model prefers grids that do not contain objects. $\lambda_{noobj}$ is to adjust the weight, so that the grids without objects have less weight. This makes the model more biased towards the loss caused by grids containing objects. Due to the existence of some small targets in this paper, the value of $\lambda_{noobj}$ needs to be reduced, and we take $\lambda_{noobj}$ equal to 0.3 in the paper.

GIoU is used to calculate the positioning loss in YOLOv5s model. Compared with IoU, GIoU can better reflect the overlap between the predicted box and the real box, but GIoU always only considers the overlap rate, which cannot well describe the regression problem of the target box^54^. When the prediction box is inside the real box, for the prediction boxes with different positions but the same size, GIoU cannot distinguish the position relationship between the prediction boxes, especially when the seeker is far from the target, and the target is smaller, which is more likely to happen. Therefore, this paper uses CIoU instead of GIoU as the loss function of target regression, and the formula is as follows:

5 $$CIoU = IoU - \frac{{\rho^{2} (b,b^{gt} )}}{{c^{2} }} - \alpha v$$

6 $$\alpha = \frac{v}{(1 - IoU) + v}$$

7 $$v = \frac{4}{{\pi^{2} }}\left( {\arctan \frac{{w^{gt} }}{{h^{gt} }} - \arctan \frac{w}{h}} \right)^{2}$$

where α is an equilibrium parameter and does not participate in gradient calculation, and v is a parameter used to measure the consistency of aspect ratio. CIoU comprehensively considers the overlap rate, the distance from the center point, and the aspect ratio between the real box and the predicted box, which makes the regression of the target box more stable and have higher convergence accuracy, and is beneficial to improving the detection accuracy of infrared small targets.

### Regression of target box

**Figure 5:**
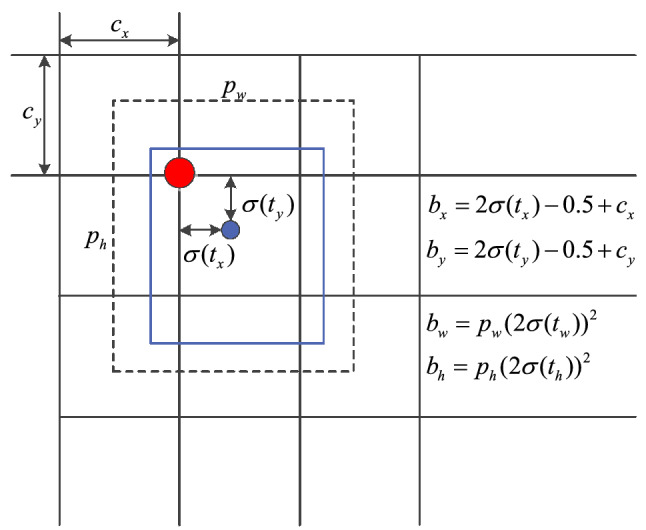
Target box regression schematic diagram.

Target box regression aims to find some mapping relationship, so that the mapping of candidate target box is infinitely close to the real target box. The prediction of the real target box is to regress the relative coordinates of the target box relative to the upper left corner of a grid by means of relative position. The relationship between the prior box and the prediction box is shown in Fig. 5, where the dashed box represents the prior box, and the solid box represents the prediction box. The prediction box is obtained by translating and scaling the prior box. The original picture is divided into S × S grid cells according to the size of the feature map, and each grid cell predicts 3 prediction boxes, each containing 4 coordinate information and 1 confidence information. When the center coordinates of a target in the real box fall in a grid, the grid predicts the target.

The coordinate prediction of the target box is calculated as follows:

8 $$b_{x} = 2\sigma (t_{x} ) - 0.5 + c_{x}$$

9 $$b_{y} = 2\sigma (t_{y} ) - 0.5 + c_{y}$$

10 $$b_{w} = p_{w} (2\sigma (t_{w} ))^{2}$$

11 $$b_{h} = p_{h} (2\sigma (t_{h} ))^{2}$$

12 $$P(object) \times CIoU(b,object) = \sigma (t_{0} )$$

where $t_{x}$, $t_{y}$, $t_{w}$, and $t_{h}$ are the offset, and $\sigma$ is the Sigmoid activation function, which is used to map the network predicted values $t_{x}$, $t_{y}$, $t_{w}$, and $t_{h}$ to [0,1]; $c_{x}$,$c_{y}$ is the offset in the cell grid relative to the upper left corner of the picture; $p_{w}$ and $p_{h}$ are the width and height of the prior box, respectively; $b_{x}$, $b_{y}$ and $b_{w}$, $b_{h}$ are the central coordinates of the prediction target box; $\delta (t_{0} )$ is the confidence of the prediction box, which is obtained by multiplying the probability of the prediction box and the CIoU value of the prediction box and the real box. A threshold for $\delta (t_{0} )$ is set, the prediction boxes with low confidence are filtered out, and then the non-maximum suppression is used. The NMS algorithm for the remaining prediction boxes is used to determine the final prediction box.

In this paper, a small target detector is added to form four detectors. On the smallest feature map, because its receptive field is the largest, it should be used to detect large targets. Therefore, the large-scale feature map should use a small-scale prior frame, and the small-scale feature map should use a large-scale prior bounding box for regression of prediction boxes. In this paper, the corresponding relationship between the feature map size of four scales and the prior bounding box size is shown in Table 1.

**Table 1:** Correspondence between the size of feature map and the size of prior frame.

Feature map size	Prior bounding box size		
80 × 80	[3, 4]	[4, 8]	[6, 7]
40 × 40	[7, 12]	[9, 15]	[12, 18]
20 × 20	[15, 27]	[23, 29]	[36, 46]
10 × 10	[97, 68]	[136, 97]	[168, 176]

## Experiment and analysis

### Infrared tank target data set

The acquisition cost of the measured infrared tank target data is high, and a large number of flight tests are needed to obtain sufficient data. The data set used in this paper includes measured infrared data and simulated infrared data. The infrared tank data set is expanded to 13,379 images by data augmentation.

**Figure 6:**
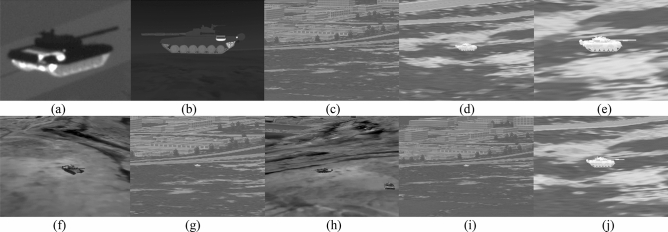
Infrared data set.

The material library of infrared simulation data includes common soil, vegetation, rocks, water, roads, building materials, general paints, and pigments. Based on the physical characteristics, the infrared target is modeled, which can reflect the corresponding spectral radiation characteristics and atmospheric transmission characteristics. The real infrared characteristics analysis and image rendering are carried out by combining the real target model, material characteristics, target thermal characteristics, and environmental thermal radiation characteristics. After the infrared scene and infrared target model are built, the infrared target imaging data of seeker can be simulated and generated by adding flight parameters. The measured data are hung by infrared seeker. Some infrared images in the data set are shown in Fig. 6. Table 2 shows the image size, number of images and target number under different ground backgrounds. Table 3 shows the number of simulated infrared images and measured infrared images, as well as the number of images in training set and verification set.

**Table 2:** Types of ground background and image data contained in infrared tank target data set.

Ground background type	Image size	Number of images	Target number
Building background	1024*768	4013	Single target
Grassland background	1920*1080	2675	Single target
Desert background	1000*1000	2943	Single target
Field background	500*500	2077	Single target
Mountain background	512*512	1725	multiple target

**Table 3:** Division of training connection and verification set in data set.

Data type	Number of images	Training set	Validation Set
Simulated infrared data	6561	5249	1312
Measured infrared data	6818	5455	1363

### Evaluation indicators

Evaluation indicators generally use the common evaluation indicators in target detection algorithms, such as mean AP, mAP, precision, and recall, to evaluate the performance of this algorithm. Precision refers to the number of correct positive samples in the prediction data set divided by the number of positive samples predicted by the model. Recall rate refers to the number of correct positive samples in the prediction data set divided by the number of actual positive samples. The above indicators are calculated as follows:

13 $$AP = \int_{0}^{1} {P{\text{d}}R}$$

14 $$mAP = \frac{{\sum\limits_{i = 1}^{N} {AP_{i} } }}{N}$$

15 $${\text{Precision = }}\frac{TP}{{TP{ + }FP}}$$

16 $${\text{Rcall = }}\frac{TP}{{TP + FN}}$$

TP, FP, and FN represent the number of correct detection boxes, false detection boxes, and missed detection boxes, respectively.

In this paper, we use mAP_0.5 and mAP_0.5:0.95 evaluation indicators. mAP_0.5 refers to calculating the average accuracy of all pictures in each category when IoU is set to 0.5, and then averaging all categories. mAP_0.5:0.95 refers to the average precision values at different IoU thresholds (from 0.5 to 0.95, with a step size of 0.05).

### Experimental results

Table 4 shows the experimental results on the typical infrared tank target data set in this paper and compares the YOLOv5s-THSE detection model proposed in this paper with the YOLOv5s, YOLOv5s + SE, and YOLOv5s + CBAM models. Here, the size of the input image is set to 640*640, the training period is set to 300 generations, the initial learning rate of training is 0.001, the learning rate is attenuated by 0.001 every 10 generations, and the batch size is set to 16. The training experiment is based on PyTorch, and the GPU is GeForce GTX 2080 Ti.

**Table 4:** Comparison of indicators of different models.

Methods	Precision	Recall	mAP_0.5	mAP_0.5:0.95
YOLOv5s	0.84216	0.73334	0.78607	0.60318
YOLOv5s + SE	0.95921	0.79243	0.84896	0.62097
YOLOv5s + CBAM	0.87298	0.78593	0.82132	0.61942
YOLOv5s-THSE	0.98848	0.87090	0.89032	0.67312

**Figure 7:**
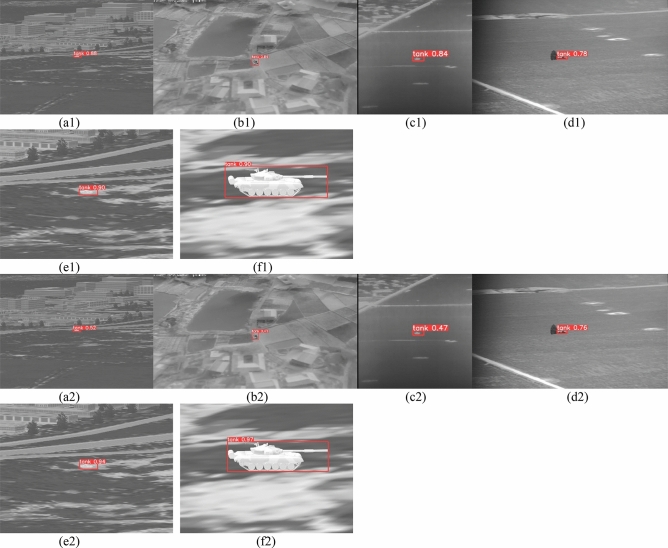

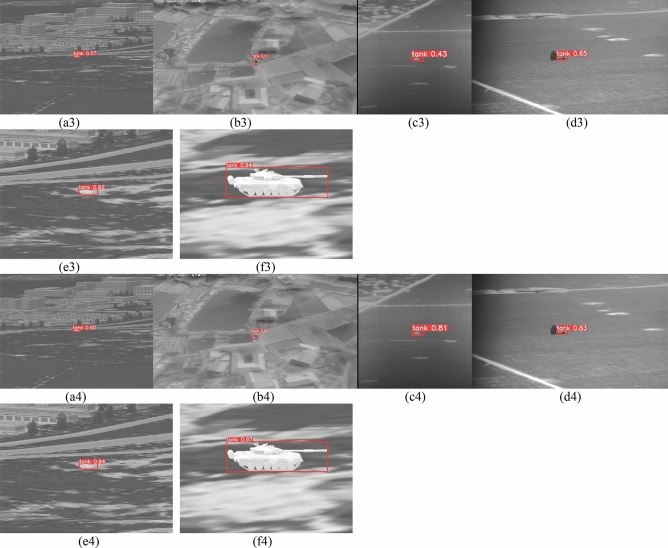
Target detection results of different models under different opposite backgrounds.

Table 4 shows that compared with the other models, the YOLOv5s-THSE proposed in this paper has evident improvements in precision, recall, mAP_0.5, and mAP_0.5:0.95. In terms of precision, YOLOv5s-THSE is 14.632%, 2.927%, and 11.550% higher than YOLOv5s, YOLOv5s + SE, and YOLOv5s + CBAM, respectively. In terms of recall, YOLOv5s-THSE is 13.756%, 7.847%, and 8.497% higher than YOLOv5s, YOLOv5s + SE, and YOLOv5s + CBAM, respectively. In terms of mAP_0.5, YOLOv5s-THSE is 10.425%, 4.136%, and 6.90% higher than YOLOv5s, YOLOv5s + SE, and YOLOv5s + CBAM, respectively. In terms of mAP_0.5:0.95, YOLOv5s-THSE is 6.994%, 5.215%, and 5.370% higher than YOLOv5s, YOLOv5s + SE, and YOLOv5s + CBAM, respectively. These data show that the model in this paper has a good detection effect on infrared tank targets under ground background.Figure 7 shows the target detection results of infrared images, including a variety of complex ground backgrounds. (a) The sequence pictures are the tank targets in the grassland background containing urban buildings. (b) The sequence pictures are the tank targets in the field background. (c) The sequence pictures are the tank targets in the grassland background. (d) The sequence pictures show the desert background tank targets with certain vegetation. (e) The sequence pictures are the scenes with gray levels similar to those of tanks. The first line is the detection result of YOLOv5s, the second line is the detection result of YOLOv5s + SE, the third line is the detection result of YOLOv5s + CBAM, and the fourth line is the detection result of YOLOv5s-THSE proposed in this paper. Here, the same four pictures are used to compare the test results.

Figure 7 shows that the detection results of YOLOv5s-THSE model adopted in this paper are better than those of the four other models in six different scenarios, and it has good detection accuracy at different scales.

**Figure 8:**
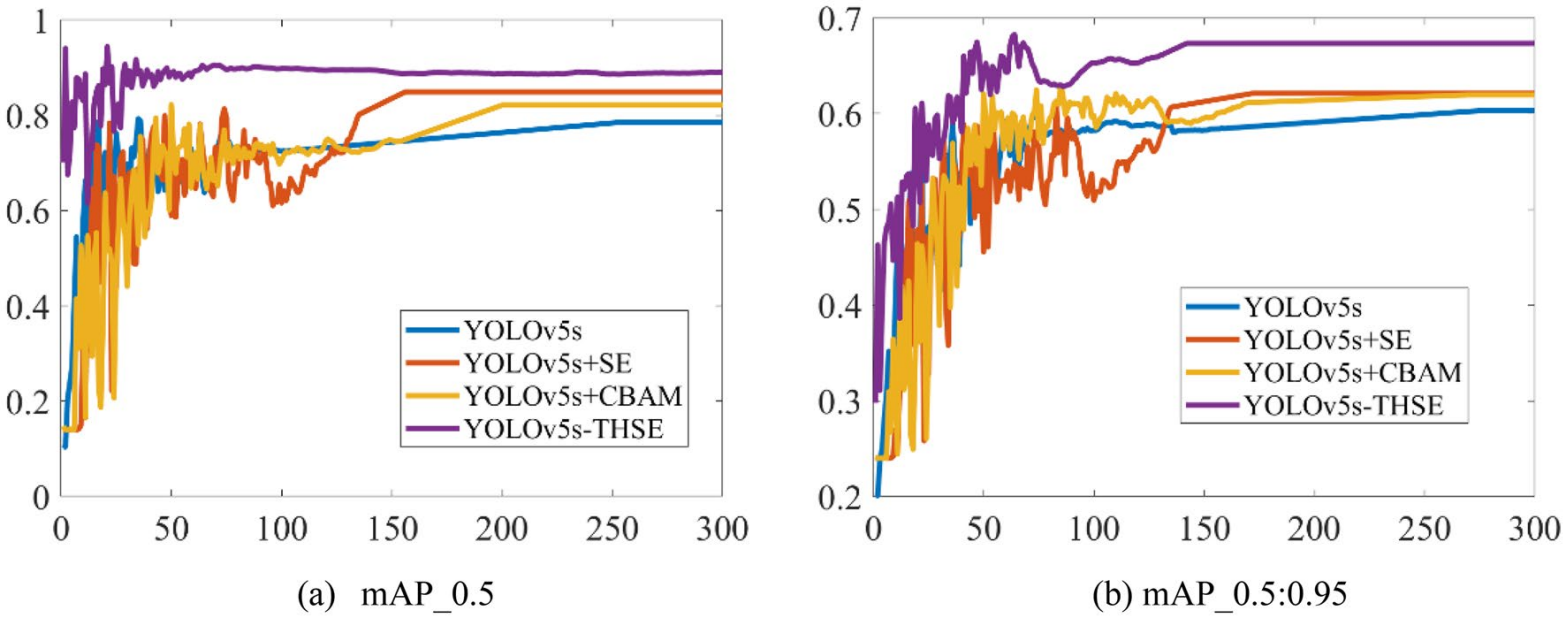
Comparison of mAP_0.5 and mAP_0.5:0.95 curves of different models, (**a**) Precision metrics, (**b**) Recall metrics.

**Figure 9:**
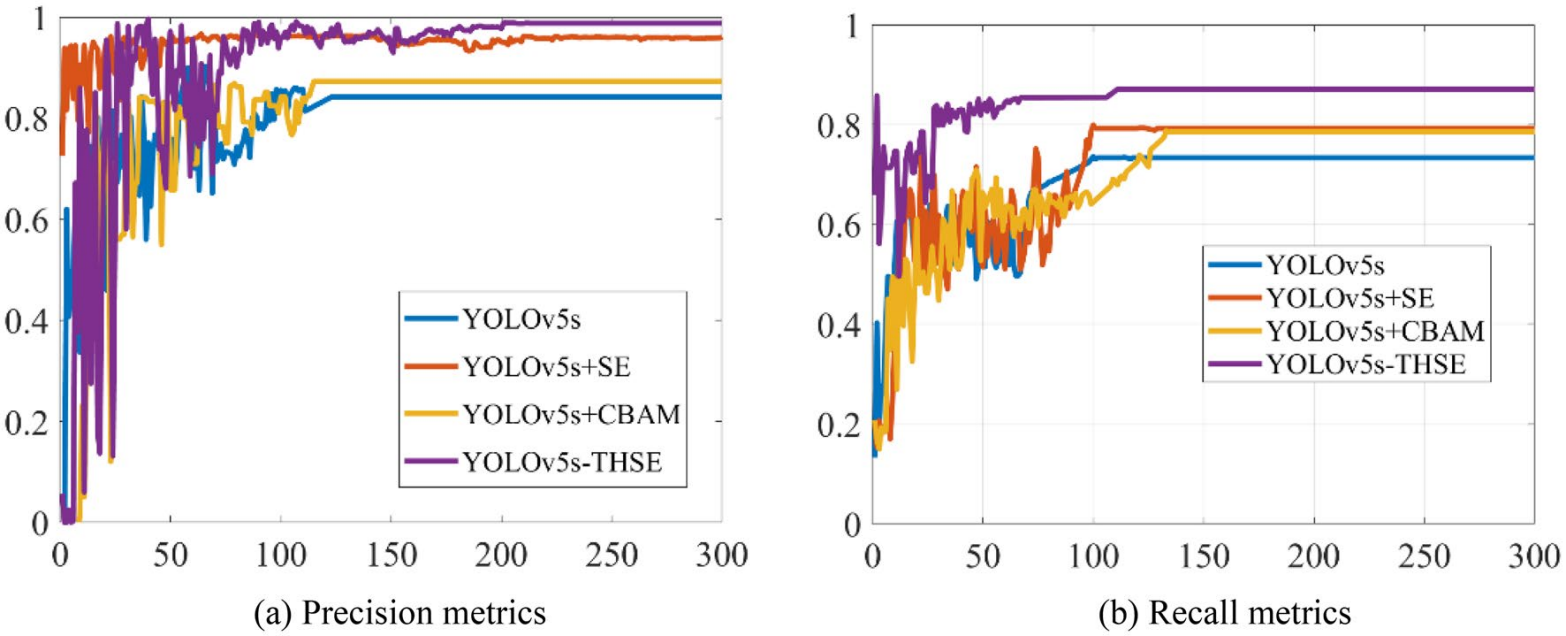
Comparison of precision and recall index of different models.

To study the index changes of different models in training, Fig. 8 shows the change trend of mAP_0.5 and mAP_0.5:0.95 in the training of several models, such as YOLOv5s-THSE, YOLOv5s, YOLOv5s + SE, and YOLOv5s + CBAM. Figure 8a shows the change of the average detection accuracy of categories when the CIoU threshold is 0.5. Figure 8b shows the change of average detection accuracy when the CIoU threshold is between 0.5 and 0.95. Compared with the YOLOv5s model, the YOLOv5s + SE model and the YOLOv5s + CBAM model add an attention mechanism, so the detection accuracy of these two methods is higher than that of the YOLOv5s model. However, the YOLOv5s-THSE model proposed in this paper adds a small target detector and a multi-head attention mechanism. The experimental results in Fig. 9 show that the YOLOv5s-THSE model has the highest precision and recall compared with the three other models, it can quickly converge to a higher precision during the training, and it takes less training period to enter a stable state.

**Figure 10:**
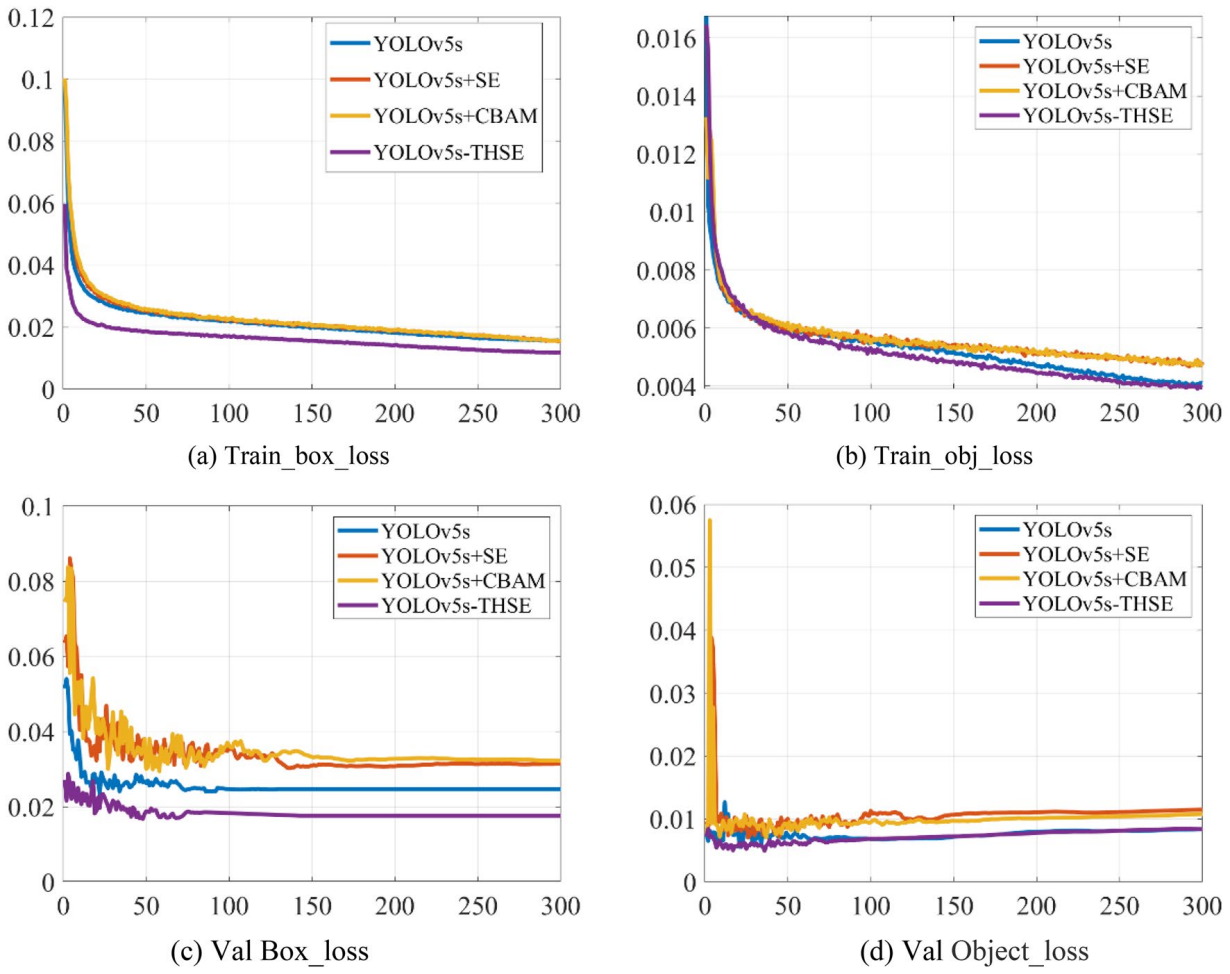
Comparison of loss function values of different models on training set and verification set. (**a**) Train_box_loss, (**b**) Train_obj_loss, (**c**) Val Box_loss, (**d**) Val Object_loss.

As the objective function of optimization in the whole training, the smaller the loss value is, the closer the detection is to the real result, and the smaller the loss value is, the better the performance of the model. Figure 10 shows the changes of several models in this paper in the training set and the verification set. Box loss and object loss on the training set show a downward trend and finally stabilize. In Fig. 10a and b, the box loss and the object loss of the YOLOv5s-THSE model decrease most rapidly, and the minimum value after stabilization is the smallest. Similarly, in Fig. 10c and d, the box loss and the object loss of the YOLOv5s-THSE model also decrease the fastest and have the smallest values. This result shows that using CIoU in the YOLOv5s-THSE model can make the detection result closer to the real target position.

To verify the improvement of small target detection by adding a small target detector in YOLOv5s-THSE model, an infrared image data set with the distance between the seeker and the target greater than 5 km is constructed. At this distance, the target pixel in the infrared image is less than 15*25. Then through data enhancement, the data set is expanded to 5000, and the above models are used for training and detection.

**Figure 11:**
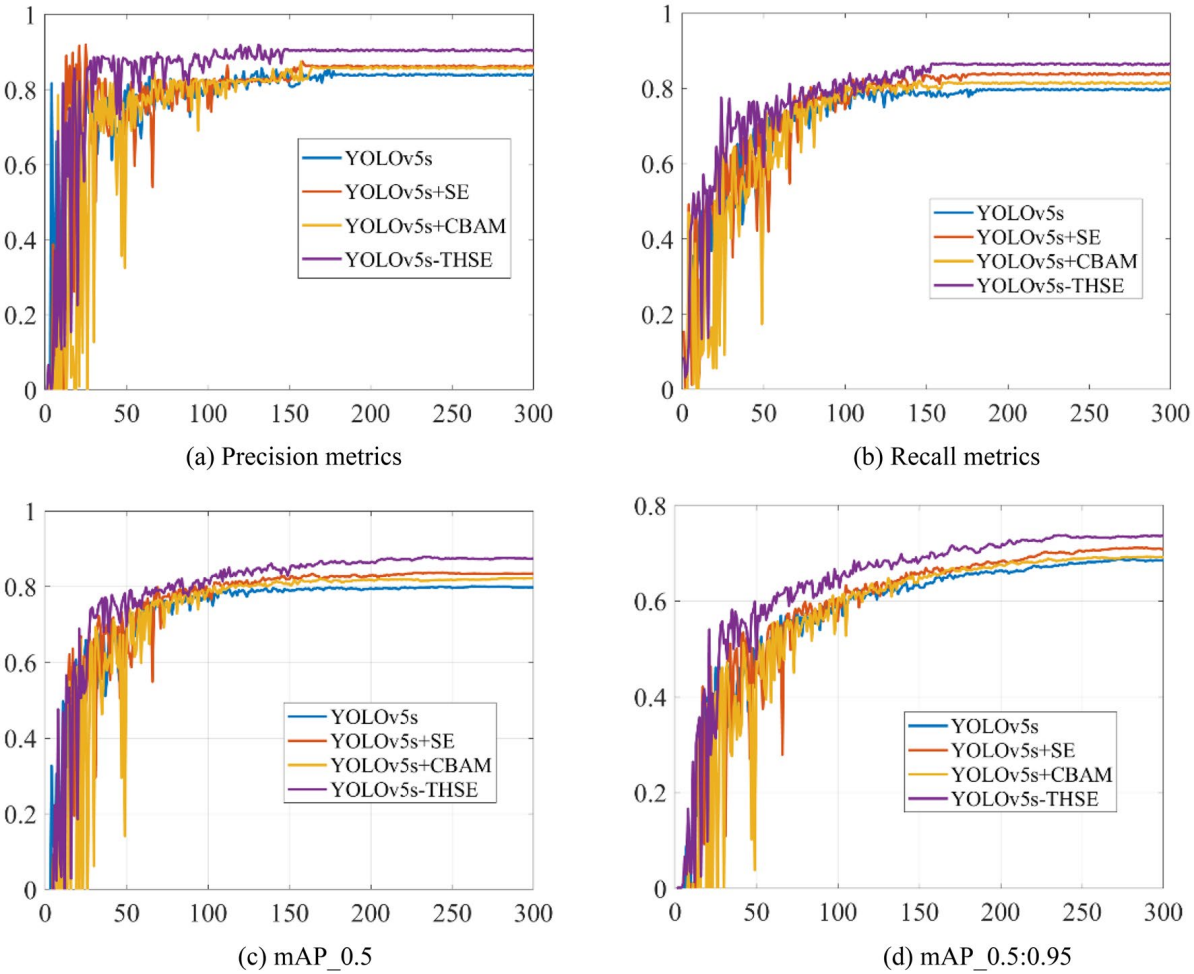
Comparison of indicators on small target data sets. (**a**) Precision metrics, (**b**) Recall metrics, (**c**) mAP_0.5, (**d**) mAP_0.5:0.95.

**Figure 12:**
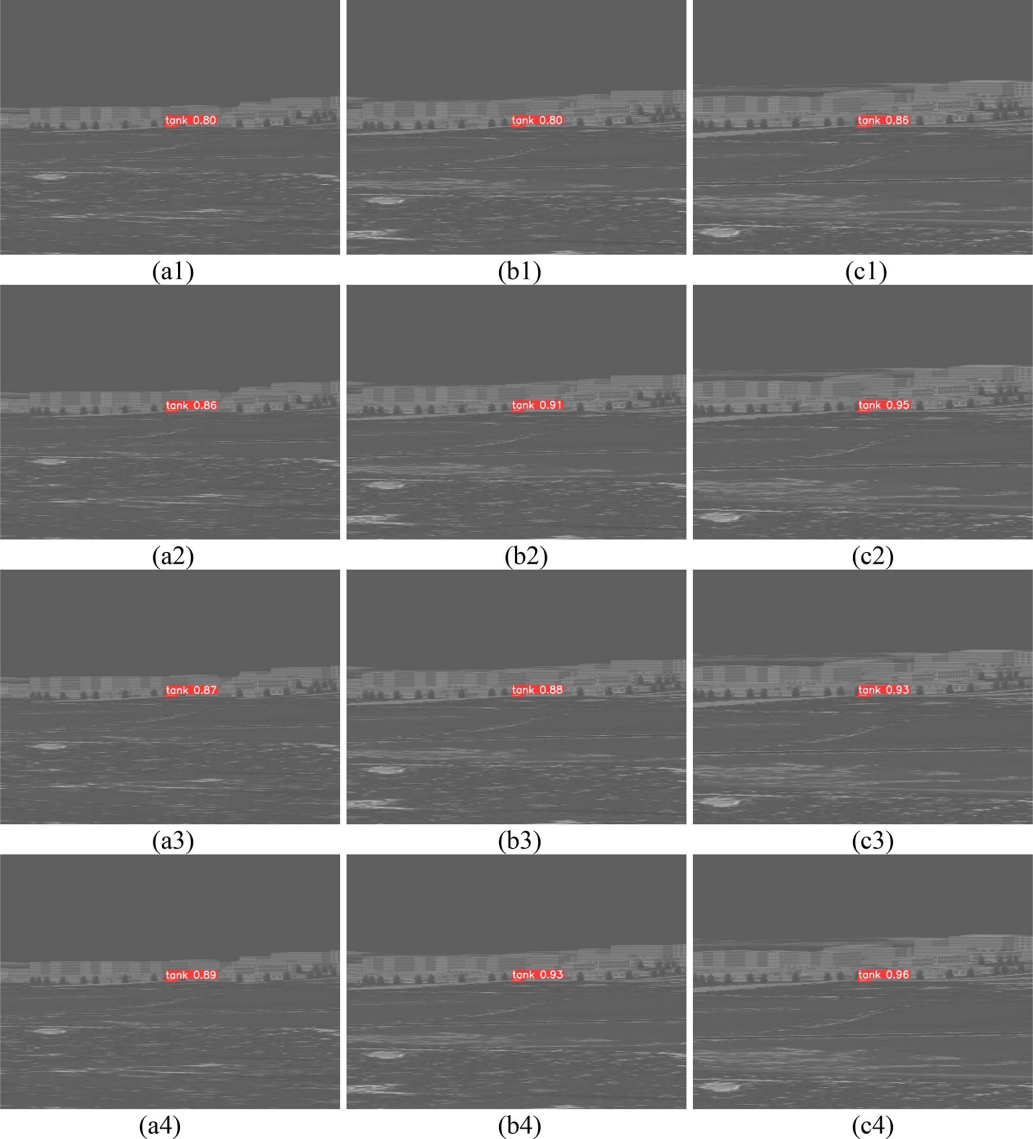
Comparison of small target detection results.

Figure 11 shows the change trend of indicators of different models on small target data, and Fig. 11a shows the change trend of precision metrics indicators in the training. The final convergence value of precision metrics of YOLOv5s-THSE model is the highest. Figure 11a shows the changing trend of recall metrics in the training. Compared with the three other models, the YOLOv5s-THSE model has the highest convergence value, less fluctuation of training curve, and fewer training iterations required for convergence. Figure 11c shows the change of average detection accuracy of categories when the CIoU threshold is 0.5. Figure 11d shows the change of average detection accuracy when the CIoU threshold is between 0.5 and 0.95. The YOLOv5s-THSE model has higher detection accuracy under these two different threshold modes. The detection results of the three groups of small targets are given in Fig. 12. In the image of group (a), the infrared seeker is 7 km away from the target, and the target size is about 5*11 pixels. In the image of group (b), the infrared seeker is 6.5 km away from the target, and the target size is about 7*13 pixels. In the image of group (c), the infrared seeker is 5 km away from the target, and the target size is about 10*15 pixels.

Table 5 compares several models for small target detection. The YOLOv5s-THSE proposed in this paper has improved precision, recall, mAP_0.5, and mAP_0.5:0.95. In terms of precision, YOLOv5s-THSE is 6.023%, 4.056%, and 4.510% higher than YOLOv5s, YOLOv5s + SE, and YOLOv5s + CBAM, respectively. In terms of recall, YOLOv5s-THSE is 6.668%, 2.355%, and 4.915% higher than YOLOv5s, YOLOv5s + SE, and YOLOv5s + CBAM, respectively. In terms of mAP_0.5, YOLOv5s-THSE is 7.564%, 3.945%, and 5.156% higher than YOLOv5s, YOLOv5s + SE, and YOLOv5s + CBAM, respectively. In terms of mAP_0.5:0.95, YOLOv5s-THSE is 5.154%, 2.809%, and 4.490% higher than YOLOv5s, YOLOv5s + SE, and YOLOv5s + CBAM, respectively. These results show that the model in this paper can improve the detection of small targets in tanks.

**Table 5:** Comparison of indicators of different models on small target data sets.

Methods	Precision	Recall	mAP_0.5	mAP_0.5:0.95
YOLOv5s	0.84216	0.79745	0.79842	0.68538
YOLOv5s + SE	0.86192	0.84058	0.83461	0.70883
YOLOv5s + CBAM	0.85738	0.81498	0.82250	0.69202
YOLOv5s-THSE	0.90248	0.86413	0.87406	0.73692

## Conclusion

Aiming at the problem of infrared tank target detection under complex ground background, this paper proposes a YOLOv5s-THSE model. After the discussion and experiments in this paper, the following conclusions can be reached: 91) the multi-head attention mechanism added in the Backbone and Neck of the network can make the network mine deeper target feature information. (2) The CSP_SE structure is added to the Neck network to suppress the background and make the model pay more attention to the target. Compared with other models mentioned in this paper, the experimental evaluation results show that introducing the multi-head attention mechanism and CSP_SE structure effectively improves the detection accuracy of infrared targets in complex ground background. 3) The small target detection Head added to the head part of YOLOv5s-THSE model can effectively improve the detection accuracy of infrared small targets, and the improvement of small target detection means that the infrared seeker has a larger detection range. On the other hand, through the experimental training curve, it can be concluded that CIoU has a smaller loss value, which is more conducive to the training and regression of object detection. These measures are of positive importance for the multiscale detection and identification of infrared seeker under complex ground. In later research, adding more measured infrared tank data will be considered to expand the data set. The model is lightweight to ensure accuracy and meet the actual use requirements.

## Data Availability

The datasets generated during and/or analysed during the current study are available from the corresponding author on reasonable request.
